# Mississippi Valley Association of Dental Surgeons

**Published:** 1848-01

**Authors:** 


*Mississippi Valley Association of Dental Surgeons.*—This Association commenced its third annual meeting in Cincinnati, September 7, 1847, and, judging from the proceedings as published in the first number of the "Dental Register," it was well attended. The opening address was read by Dr. E. Taylor, president of the Association, and contains many important and valuable suggestions on the importance of high professional attainment. It should be read by every member of the profession. He points out in a clear and lucid manner the mutual obligations of the members to each other. Having banded together for the purpose of mutual instruction, each is bound to contribute all in his power to the common fund of professional knowledge, as the whole is made available to him, and Dr. T. would make it his proud ambition to have the largest possible amount of capital invested in this fund. He urges this importance of correct practical information, and as correct theory is necessary to this, he incidentally glances at the branches of knowledge that should enter into the education of the practitioner of dental surgery. Dental empiricism finds no favor with Dr. T. and to a certain class, he deals some very keen thrusts, but we fear his prediction, that as they, "like Jonah's gourd" "grow up in a night" will "perish in a night," is not likely immediately to be fulfilled. If we had room, we would publish the opening address entire, but as we cannot do this, we would advise such of our readers as may be desirous of procuring it to subscribe for the Dental Register.

Dr. A. Berry, read an interesting address on tobacco, in which he briefly notices the properties and effects of this herb upon the animal economy. An address was also read at the same meeting by Dr. Rogers, on "Nerve Killing," and another by Dr. J. Allen, on the contour of the face.

We should be glad to notice the proceedings of the Association more at length, but we have not time to do so at present. The officers for the ensuing year, are Wm. B. Ross, president; W. E. Ide, D. J. Hunt and B. B Brown, first, second and third vice-presidents; J. Allen, recording secretary; James Taylor, corresponding secretary; and C. Bonsall, treasurer; examining committee, W. H. Goddard, M. Rogers and Edward Taylor; executive committee, S. Griffith, A. Berry and J. Allen, edititing and publishing committee, S. P. Hullihen, B. B. Brown and James Taylor.

The subjects of the addresses for the next annual meeting which is to be held in Cincinnati the first Tuesday in September, 1848, are black teeth, plugging teeth, extracting teeth, irregularity of the teeth, cleanliness of the mouth, and pivot teeth. Dr. B. B. Brown is appointed to deliver the opening address.—*Balt. Ed.*

